# Discovering Myeloid Cell Heterogeneity in Mandibular Bone – Cell by Cell Analysis

**DOI:** 10.3389/fphys.2021.731549

**Published:** 2021-09-30

**Authors:** Kyu Hwan Kwack, Natalie A. Lamb, Jonathan E. Bard, Elliot D. Kramer, Lixia Zhang, Scott I. Abrams, Keith L. Kirkwood

**Affiliations:** ^1^Department of Oral Biology, University at Buffalo, The State University of New York, Buffalo, NY, United States; ^2^Genomics and Bioinformatics Core, New York State Center of Excellence in Bioinformatics and Life Sciences, University at Buffalo, The State University of New York, Buffalo, NY, United States; ^3^Department of Biochemistry, Jacobs School of Medicine and Biomedical Sciences, University at Buffalo, Buffalo, NY, United States; ^4^Department of Medicine, University at Buffalo, Buffalo, NY, United States; ^5^Department of Immunology, Roswell Park Comprehensive Cancer Center, Buffalo, NY, United States; ^6^Department of Head and Neck, Plastic and Reconstructive Surgery, Roswell Park Comprehensive Cancer Center, Buffalo, NY, United States

**Keywords:** hematopoietic progenitor cell (HPCs), myeloid cell, transcriptome, bone marrow, cellular microenvironment, mandible

## Abstract

The myeloid-derived bone marrow progenitor populations from different anatomical locations are known to have diverse osteoclastogenesis potential. Specifically, myeloid progenitors from the tibia and femur have increased osteoclast differentiation potential compared to myeloid progenitors from the alveolar process. In this study, we explored the differences in the myeloid lineage progenitor cell populations in alveolar (mandibular) bone versus long (femur) bone using flow cytometry and high-throughput single cell RNA sequencing (scRNA-seq) to provide a comprehensive transcriptional landscape. Results indicate that mandibular bone marrow-derived cells exhibit consistent deficits in myeloid differentiation, including significantly fewer myeloid-derived suppressor cell (MDSC)-like populations (CD11b^+^Ly6C^+^, CD11b^+^Ly6G^+^), as well as macrophages (CD11b^+^F4/80^+^). Although significantly fewer in number, MDSCs from mandibular bone exhibited increased immunosuppressive activity compared to MDSCs isolated from long bone. Using flow cytometry panels specific for bone marrow progenitors, analysis of hematopoietic stem cells showed no defects in mandibular bone marrow in LSK (Lin^–^Sca1^+^cKit^+^) cell and LK (Lin^–^Sca1^–^cKit^+^) cell populations. While there was no significant difference in granulocyte progenitors, the granulocyte-monocyte progenitors and monocyte progenitor population were significantly decreased in the mandibular bone marrow. T-lymphocyte subsets were not significantly different between mandibular and femoral bone, except for CD4^+^CD25^+^Foxp3^+^ regulatory T lymphocytes, which were significantly increased in the mandible. In addition, B lymphocytes were significantly increased in mandible. Single cell RNA sequencing from mandible and femur BM revealed distinct differences in transcriptomic profiles in myeloid populations establishing previously unappreciated aspects of mandibular bone marrow populations. These analyses reveal site-specific differences in the myeloid progenitor cellular composition and transcriptional programs providing a deeper appreciation of the complex differences in myeloid cell heterogeneity from different anatomical bone marrow sites.

## Introduction

Bone mass and shape is continuously adapting to variations in load caused by physical activity, mechanical force, hormones, nutrients, and several additional osteotropic signaling molecules. The adult skeleton is a highly specialized and dynamic organ that undergoes a constant and cyclic bone remodeling process where old bone is removed by bone resorption followed by new bone formation – a process essential for skeleton health maintenance. This sequential process requires bone-forming osteoblasts and bone-resorbing osteoclasts, which in the case of osteoclasts demand a constant pool of progenitor populations. Currently, it has been appreciated that these the bone marrow progenitor cells not only differ depending on anatomic location, but also these progenitor cells respond differently to biological signals (Faloni et al., 2011). Thus, understanding bone marrow progenitor cellular populations may provide new insights into their site-specific function.

Alveolar bone differs morphologically and functionally from another skeletal bones. The adult alveolar bone remodels more rapidly than any other skeletal bones (Huja et al., 2006). This bone turnover distinction is most likely due to the fact that the alveolar bone arises from the neural crest cells of the neuroectoderm germ layer, not from the mesoderm (Chai and Maxson, 2006). Moreover, bone ossification is different in the alveolar process compared to other bones, which proceeds by intramembranous rather than endochondral ossification (Karaplis, 2002). In addition, occlusal stress stimulation and tooth-derived inflammatory responses, which exist only in the alveolar bone, affects metabolism as well as remodeling (Huja et al., 2006; Gruber, 2019; Lerner et al., 2019; Connizzo et al., 2021). The clinical significance of these differences can be appreciated from skeletal diseases that have a greater predilection for the alveolar bone, including hyperparathyroid jaw tumor syndrome, cherubism and osteonecrosis associated with anti-resorptive therapeutics, including bisphosphonates (Ueki et al., 2001; Simonds et al., 2002; Ruggiero et al., 2004). Likewise, there are conditions in which the skeletal bone is affected more than the alveolar process. In the case of malnutrition and ovariectomy, mandibular bone decreases bone mineral density in the trabecular compartment at a lower rate than what is observed in the tibia (Mavropoulos et al., 2007). Additional differences have been observed osteogenic potential where alveolar-derived bone marrow stromal cells or mesenchymal stem cells show exhibit higher osteogenic potential compared to other skeletal bones (Matsubara et al., 2005; Akintoye et al., 2006; Aghaloo et al., 2010; Damek-Poprawa et al., 2010; Yamaza et al., 2011). Thus, fundamental differences exist between mandibular bone and other long bones leading to different physiological and pathological responses and clinical presentations. Consequently, due to the specificity of the mandibular bone and the ease of access cells from long bone versus mandibular bone, there is a relative paucity of data focused on cellular differences between mandibular/oral and long bone.

The bone marrow derived cellular populations that play a major role in bone remodeling are osteoblasts that help generate a mineralized matrix and osteoclasts that resorb this matrix (Crockett et al., 2011). The osteoclastogenic populations are derived with the hematopoietic progenitor stem cell lineage. However, the myeloid progenitor cells are osteoclastogenic precursors that differentiate into highly diverse populations. Mononuclear myeloid cells include monocytes as well as macrophages, dendritic cells (DC) and osteoclasts that terminally differentiate in tissues under physiologic and inflammatory conditions alike. Granulocytic myeloid cells contain populations of eosinophils, basophils, polymorphic nuclear neutrophils, and mast cells. The proliferation and differentiation of myeloid progenitor cells in the bone marrow is strictly controlled according to the physiological and pathological conditions. Hematopoietic stem cells (HSCs) reside in the bone marrow, maintaining a pool of pluripotent stem cells through self-renewal, or progenitor cellular populations capable of differentiation into committed lineages (Seita and Weissman, 2010). The HSC population can be identified based on expression of cell surface markers. Accordingly, HSC/progenitor cells are negative for mature hematopoietic markers (Lineage markers) and positive for the stem cell markers Sca-1 and c-Kit (Lineage^–^Sca-1^+^c-Kit^+^; LSK) (Challen et al., 2009). The myeloid progenitor population downstream of HSC, can be identified as a Sca-1 negative subset of the lineage negative and c-Kit positive population (Lineage^–^Sca-1^–^c-Kit^+^; LK). This fraction is called hematopoietic progenitor cells (HPCs) and can be further divided into subtypes of common myeloid progenitors (CMPs), megakaryocyte-erythroid progenitors (MEPs), and granulocyte-macrophage progenitors (GMPs). Myeloid-derived suppressor cells (MDSCs) are a heterogenous population of myeloid-cell lineage consisting of precursors of myeloid cells and myeloid-cell progenitors. MDSCs include two relatively well characterized subtypes of monocytic (M)-MDSCs and granulocytic (PMN)-MDSCs. In mice, M-MDSC can be defined as CD11b^+^Ly6G^–^Ly6C^*high*^ and PMN-MDSC as CD11b^+^Ly6G^+^Ly6C^*low*^. These cells have the ability to modulate the innate and adaptive immune response. Under pathologic conditions, the immature myeloid cells (IMCs) generated in the bone marrow are partially blocked from differentiation into mature myeloid cells, resulting in the expansion of the IMC population. And the prolonged and marked expansion of the IMCs lead to the expansion of MDSC population (Gabrilovich and Nagaraj, 2009). Several reports support MDSC plasticity as osteoclast progenitors under various pathological conditions associated with bone destruction (Sawant et al., 2013; Zhang et al., 2015; Kirkwood et al., 2018). Therefore, to study whether the specificity of mandibular bone is due to the heterogeneity of bone marrow cells, it is necessary to explore not only the populations of lineage committed bone marrow cells but also their progenitors.

Single cell RNA sequencing (scRNA-seq) provides a comprehensive transcriptional environment for analyzing tissue heterogeneity at the individual cell level and exploring the contribution of various cell subtypes to physiological function. To date, there are no studies directly comparing the transcriptomic landscape between mandibular and long bone marrow. In this study, we analyzed the heterogeneity of myeloid cells in the mandibular bone and the femur by performing flow cytometry and scRNA-seq. We have found that not only these progenitor cells, but also myeloid cells have distinct transcriptomic profiles.

## Materials and Methods

### Animals

Animal maintenance and experimental protocols were conducted under an approved Institutional Animal Care and Use Committee protocol from the University at Buffalo using ARRIVE guidelines. Male C57BL/6J mice were purchased from The Jackson Laboratory (Bar Harbor, ME, United States) at 12 weeks of age. All animals were housed in an environment-controlled conditions of 22 ± 2°C, 40–70% humidity with a light/dark cycle of 12 h.

### Bone Marrow Cell Isolation

Soft tissue was dissected from 3-month-old C57BL/6 mouse femurs and mandibles. Long-bone BM cells were flushed from the femurs with RPMI 1640 (Corning Inc., Corning, NY, United States) medium. To obtain the bone marrow cells from mandible, cut the anterior bone along the mental foramen at the mesial of the first molar. Mandibular ramus, including the angular, condylar and coronoid process, was removed along the distal edge of the third molar on the distal side to expose the bone marrow cavity. Bones were then placed in 1.5 ml microfuge tubes supported by 0.5 ml microfuge tube inserts with a lower hole, and centrifuge at 8,000 rpm for 10 min. The bone marrow pellet was resuspended 5 ml of RPMI 1640 culture medium. A single cell suspension was obtained by passing 18-, 21-, and 25-gauge needles in sequence.

### Flow Cytometry Analysis

The isolated bone marrow cells were depleted of red blood cells using RBC lysis buffer (Gibco, Invitrogen, United States) and filtered through 70 μm nylon membrane (BD) to make a single cell suspension. Cells were suspended in staining buffer (dPBS + 0.5% BSA + 2 mM EDTA) and treated with Mouse Fc block (BD Biosciences). To characterize the phenotype of bone marrow cells, we stained for various anti-mouse: anti-Ly6C FITC (REA 796), anti-Ly6G PE (REA 526), anti-F4/80 PE-Vio770 (REA 126), anti-CD11b APC (M1/70 15.11.5), anti-CD4-Vioblue (REA 604), anti-CD8-APC-Vio770 (REA 601), anti-CD25-PE-Vio770 (7D4), anti-Foxp3-PE (REA 788), anti-CD19-FITC (6D5), anti-NK1.1-PE (PK136), anti-CD11c-APC (REA 754). Intracellular staining for Foxp3 was performed using Fixation/Permeabilization Solution Kit (BD Biosciences). Stained cells were collected on MACSQuant System (Miltenyi Biotec) and analyzed with FlowJo software (version 10.6).

For the analysis of bone marrow progenitor cells, the following directly conjugated Abs were used for staining: anti-CD16/32 PerCP-Cy5.5 (clone 93), anti-cKit APC/Fire 750 (2B8), anti-CD150 BV605 (TC15-12F12.2), anti-Ly6C Ax700 (HK1.4), anti-Sca1 PECy7 (D7), anti-Flit3 PE (A2F10), anti-CD115 APC (AFS98), anti-Lineage Cocktail Pacific Blue (17A2; RB69C5; RA36B2; Ter119; M1/70), anti-CD105 PeCy5 (MJ7/18). Stained samples were then treated with DAPI (Thermo Fisher Scientific) for dead cell exclusion. Samples were acquired on the LSR II flow cytometer (BD Biosciences) via FACSDiva version 6.1.3 software. Data analysis was performed using FCS Express 7.0 following the bone marrow progenitor markers described in Netherby et al. (2017).

### T Cell Proliferation Assay

Monocytic-Myeloid-derived suppressor cells were isolated from the femur/mandible bone marrow using the Myeloid-Derived Suppressor Cell Isolation Kit (Miltenyi Biotec) according to the manufacturer’s instruction. Total T cells were separated from the spleen using the CD3ε MicroBead Kit (Miltenyi Biotec) accordance of the manufacturer’s protocol. Isolated T cells were labeled with CTV (Invitrogen) and plated in a round-bottom 96-well plate at the density of 2 × 10^5^ cells/well. The plated T cells were activated by CD3/CD28 (Thermo Fisher Scientific), and co-cultured with the isolated M-MDSCs. After 3 days, the cells were collected, treated with Fc block and stained with antibodies as follows: CD3-APC (REA641, Miltenyi), CD4-pE-cy7 (GK1.5, eBioscience), and CD8-APC-cy7 (REA601, Miltenyi). Data were collected on MACSQuant System (Miltenyi Biotec) and analyzed with FlowJo 10.6 software.

### Single-Cell RNA Sequencing

For detailed scRNA-seq characterization, bone marrow cells isolated from the femur and mandible of the same mouse were obtained and used in these analyses. Cell suspensions were sequenced on the 10x genomics chromium platform. Following library preparation according to manufacturer protocol, libraries were loaded on an Illumina NextSeq in high-output mode, with a general target of 30,000 reads per cell to provide for sufficient depth and transcriptomic saturation. Post sequencing, the data was demultiplexed and provided as input into the 10X Genomics Cell Ranger pipeline (version 5), which quantified the transcriptomic profile of each cell against a reference genome. Sequence saturation, barcodes detected per cell, percent of transcripts in cell, and general alignment statistics were evaluated for quality. Cell Ranger matrix files were then used as input into the R Bioconductor package Seurat version 4 (Stuart et al., 2019).

Mapping rates to the mouse reference genome were above 95% for both samples. Approximately 16,140 cells from the femur and 14,338 cells from the mandible were sequenced. Cells with outlier-status, abnormal gene detection rates (<200 and >6,000), and high mitochondrial transcript load (>15%), which is an indicator of cellular stress, were filtered from the analysis (Supplementary Figure 1). After filtering, the data underwent Seurat normalization, followed by principal component analysis (PCA) and Uniform Manifold Approximation and Projection (UMAP) dimensionality reduction, and the construction of a Shared Nearest Neighbor (SNN) graph to cluster cells with similar transcriptomic profiles.

### Cluster Generation and Labeling

Both samples (femur and mandible) were integrated and run through the Seurat pipeline for guided clustering, identifying 19 distinct clusters. Clusters were first annotated through an automated platform for identifying cell types called scCATCH (Shao et al., 2020). scCATCH provided a foundation for identifying cell types, followed by manual review of marker gene on each individual cluster. To identify cell-types of interest that exist as smaller populations within the total bone marrow, individual clusters were isolated, and subjected to further sub clustering. Two initial myeloid populations were identified, which through manual review of expressed markers, were annotated into the populations of GMPs, CMPs, MEPs, and mesenchymal stem cells in total bone marrow.

### M-MDSC and PMN-MDSC Composite Score Analysis

Due to the low frequency of MDSCs in bone marrow, particularly under wildtype conditions, a composite gene scoring approach was utilized to define both M-MDSCs and PMN-MDSCs. Gene panels from M-MDSC and PMN-MDSC transcriptomic signatures previously determined through scRNA-seq were used to define these cell types (Alshetaiwi et al., 2020). To identify M-MDSCs, which are even more scarce in total bone marrow. The M-MDSC population was defined as the monocytic cluster with the highest expression of the M-MDSC gene panel (*Cxcr2, S100a9, S100a8, Ifitm1, Lrg1, Stfa2l1, Retnlg, Il1b, BC100530*, and *Gm5483*). PMN-MDSCs show similar gene expression profiles to neutrophils and thus the two cell types are grouped together in UMAP (Alshetaiwi et al., 2020). To identify PMN-MDSCs, the neutrophil cluster was subset and new clusters were then called. The PMN-MDSC cluster was identified based on expression of the PMN-MDSC gene panel (*Cd84, Ctsd, Arg2, Pla2g7, Il1b, Clec4e, Il1f9, Junb, Wfdc17, Clec4d, BC100530, Ifitm1, Dusp1, Socs3, Ccl6, Srgn*, and *Cxcr2*).

### Statistical Analysis

Flow cytometry experiments were analyzed using GraphPad Prism 8.4 (GraphPad Software Inc., La Jolla, CA, United States) with two-tailed unpaired Student’s *t* test. For scRNA-Seq, per-cell gene expression measurements were log normalized using a scale factor of 10,000 using the Seurat function NormalizeData. Cluster-to-cluster differential expression testing was preformed using the Wilcoxon Rank Sum test, using Seurat’s FindMarkers and FindAllMarkers functions requiring at least a 0.25 log2 fold change and minimum cluster participation of 10%. Heatmap and DotPlot visualization utilized Seurat’s data scaling function, centering mean gene expression values to 0 per-cell and scaling the expression of each gene per-cell to equal variance of 1.

## Results

### Mandibular Bone Marrow-Derived Cells Exhibit Consistent Deficits in Myeloid Differentiation

**FIGURE 1:**
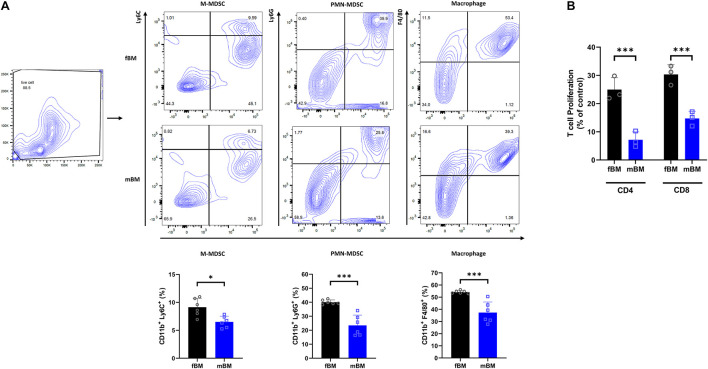
Myeloid population of mBM exhibits consistent deficits compared to fBM. **(A)** Gating strategy (Top) and calculated frequencies (Bottom) of immune cell population from fBM and mBM by flow cytometry. **(B)** T cell proliferation assay of MDSCs isolated from fBM and mBM. The graph of the statistical analyses is presented. Unpaired *t* test; data are presented as mean ± SEM of value from four independent experiments from fBM and mBM, ^∗^*P* < 0.05, ^∗∗∗^*P* < 0.001.

To determine whether the mandibular bone marrow-derived cells (mBM) differ from the femoral bone marrow-derived cells (fBM), we first confirmed the myeloid population in each bone marrow cell by flow cytometry (Figure 1A). The frequency of M-MDSC (CD11b^+^Ly6G^–^Ly6C^*high*^), PMN-MDSC (CD11b^+^Ly6G^+^Ly6C^*low*^), and macrophage (CD11b^+^F4/80^+^) were consistently lower in mandibular bone marrow-derived cells (Figure 1A). Next, we performed a T cell proliferation assay to confirm MDSC functional phenotype (Bronte et al., 2016). As shown in Figure 1B, both MDSCs isolated from fBM and mBM showed the ability to inhibit T cell proliferation, confirming that they were genuine MDSCs. Unexpectedly, although the percentage of MDSC in mBM was lower than that of fBM, MDSCs from the mandible exhibited a much greater capacity to inhibit both CD4^+^ and CD8^+^ T cellular proliferation (Figure 1B).

### Monocyte Progenitors Are Significantly Reduced in Mandibular Bone Marrow

**FIGURE 2:**
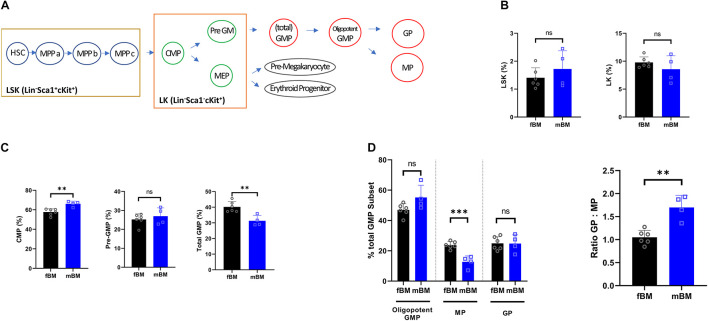
Myeloid progenitors show a decrease in the total GMP population in mandibular bone. **(A)** Schematic view for mouse HSC hierarchy. **(B)** Percentages of LSK (Lin^–^Sca1^+^cKit^+^) or LK fractions (Lin^–^Sca1^–^cKit^+^) from the bone marrow of same subject-matched fBM and mBM. **(C)** Calculated frequencies of CMPs, Pre-GMPs, and total GMPs from bone marrow of same subject-matched fBM and mBM. **(D)** Percentage of GPs, MPs and oligopotent GMPs within the total GMP population (Left). Ratio of GP to MP in fBM and mBM (Right). Unpaired *t* test; data are presented as mean ± SEM of value from four independent experiments from fBM and mBM, ^∗∗^*P* < 0.01, ^∗∗∗^*P* < 0.001.

Next, flow cytometry was employed using an antibody panels specific to bone marrow progenitor populations to determine the difference in the composition of the direct antecedents to differentiated myeloid cell populations. A schematic diagram of the mouse HSC hierarchy is shown to facilitate data presentation (Figure 2A). The LSK (Lin^–^Sca1^+^cKit^+^) and LK (Lin^–^Sca1^–^cKit^+^) populations showed no overall significant difference between fBM and mBM (Figure 2B). The total GMP subset of LK cells contains both the lineage committed granulocyte and monocyte progenitors, as well as their still oligopotent-GMP parent population (Yanez et al., 2015). This oligopotent-GMP is the bifurcation point between granulocytic and monocytic differentiation. Although there was no significant difference in the LK population as a whole, there was a significant decrease in total GMPs and an increase in CMP in the mBM compared to the fBM (Figure 2C). To further explore the difference in total GMPs, we next checked the relative frequencies of the distinct populations downstream of the total GMP. As shown in Figure 2D, MPs significantly decreased in mBM while GPs and oligopotent GMPs were unaffected. Thus, the decrease in MPs seen in mBM appears to account for most of the observed reduction in the classically defined total GMP population. To reflect further on how the total GMP response to the monocytic lineage was skewed in mBM, the ratio of GPs to MPs was calculated, which significantly increased in mBM compared to fBM (Figure 2D). Collectively, these data indicate that the decrease in total GMPs was mainly due to the reduction of the MP subsets, revealing an early instance of myelopoietic deregulation within the mBM monocytic lineage.

### Regulatory T Cells and B Cells Appear Significantly More in Mandibular Bone Marrow-Derived Cells

**FIGURE 3:**
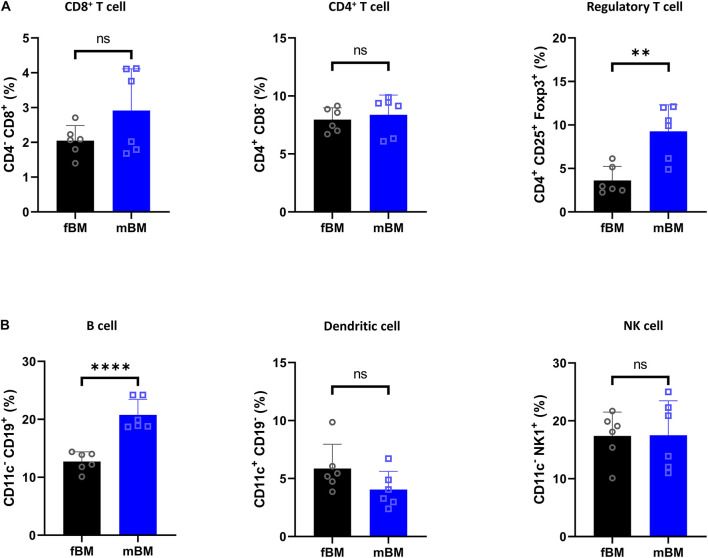
Regulatory T cell and B cells are present in higher proportions in mandibular bone. **(A)** Flow cytometry of T cell subpopulation from fBM and mBM. **(B)** Percentage of B cell, dendritic cell and NK cell from fBM and mBM. Unpaired *t* test; data are presented as mean ± SEM of value from four independent experiments consisting fBM and mBM, ^∗∗^*P* < 0.01, ^****^P < 0.0001.

We next investigated the lymphocytic and dendritic population (Figure 3). There was no significant difference in CD4^+^ T cells and CD8^+^ T cells, while there was a significant elevation of regulatory T cells in mBM (Figure 3A). In addition, there were no defects in dendritic cells (CD11c^+^CD19^–^) and natural killer (NK) cells (CD11c^–^NK1.1^+^), but B cells (CD11c^–^CD19^+^) were significantly increased in mBM compared to fBM (Figure 3B).

### Characteristics of Bone Marrow Single Cell Atlas

**FIGURE 4:**
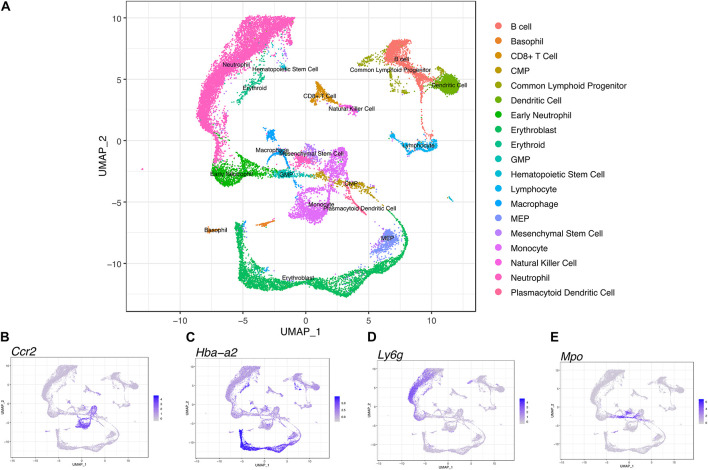
Transcriptional profiling of bone marrow-derived cells reveals a differentiation landscape with 19 distinct cell subpopulations. **(A)** Integrated Seurat analysis of femur and mandible samples from total mouse bone marrow displayed in UMAP. Clusters were annotated based on known marker genes. Feature plots of **(B)** *Ccr2*, **(C)** *Hba-a2*, **(D)** of *Ly6g*, and **(E)** *Mpo* expression shown as representative markers of monocytes, erythroblasts, neutrophils, and myeloid progenitor populations, respectively.

We performed scRNA-seq to identify cell populations and their transcriptomic signatures in both fBM and mBM. Through manual marker gene analysis, paired with the automated annotation via scCATCH, 19 distinct cell subpopulations were identified in total bone marrow (Figure 4A). Two initial clusters were merged into one monocytic population, based on *Ccr2* expression, in addition to other marker genes (Figure 4B). Cell cycle analysis also informed monocyte cluster annotation (Supplementary Figure 2), as this group contained cells in all three phases of the cell cycle. With this approach, erythroblasts based on *Hba-a2* expression (Figure 4C) and neutrophils based in *Ly6g* expression (Figure 4D) were defined. Myeloid progenitor populations were initially identified by *Mpo* expression (Figure 4E) and then further divided into MEPs, CMPs, and GMPs based on their transcriptomic signature (Paul et al., 2016), paired with scCATCH. Additional markers were used to define and annotate the remaining clusters within the UMAP (Supplementary Figure 3).

### Comparisons at the Single-Cell Level of Femoral Bone Marrow-Derived Cells and Mandibular Bone Marrow-Derived Cells

**FIGURE 5:**
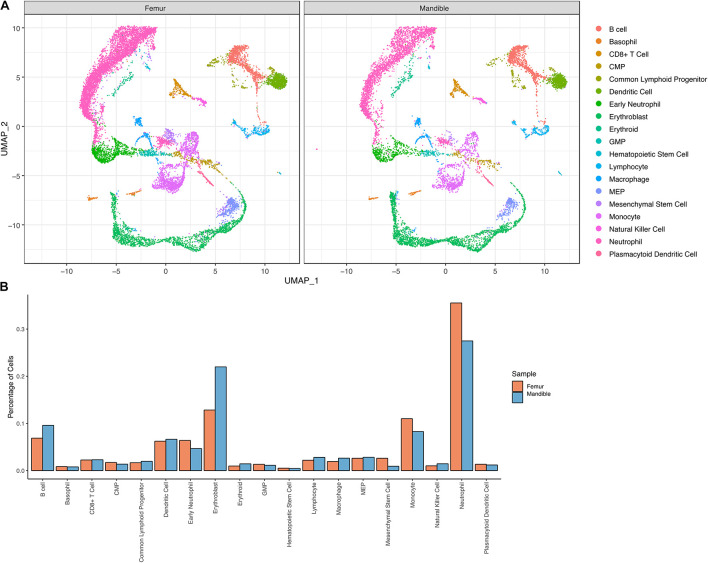
Transcriptional profiling of bone marrow-derived cells reveals slightly distinct differentiation landscapes in fBM and mBM. **(A)** UMAP of femur and mandible samples displayed individually. **(B)** The percentage of cells in each annotated cluster for both the femur (orange) and mandible (blue), plotted as a percentage of total cells sequenced in each sample.

**FIGURE 6:**
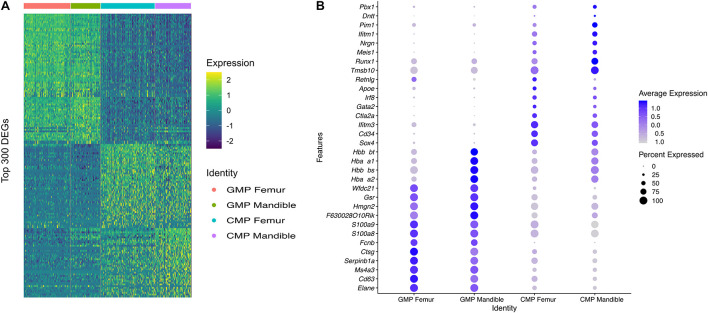
CMP and GMP populations show transcriptomic differences in fBM and mBM. **(A)** Heatmap of the topmost differentially expressed genes in both CMP and GMP populations, as well as between fBM and mBM. **(B)** DotPlot showing the top genes that are differentially expressed between the femur and the mandible in both CMP and GMP populations.

To investigate heterogeneity between fBM and mBM at the level of individual cells, in an unbiased manner, we analyzed both cell population and transcriptomic differences between the two samples. There are notable differences between the distribution of femoral and mandibular bone marrow as seen in UMAP (Figure 5A). To accurately compare the differences seen in UMAP, the percentage of each cell population in the fBM and mBM sample were determined (Figure 5B). As shown by flow cytometry, B cells were increased in the mBM, while monocytes and neutrophils were decreased in the mBM (Figure 5B). However, contrary to the flow cytometry results, the total number of CMP populations reduced in the mouse mBM compared to the fBM (Figure 5B). To examine the CMP and GMP population in more detail, transcriptomic differences were examined (Figure 6). As a result of flow cytometry analysis showed differences in the distribution of CMP and GMP populations, CMP and GMP populations also revealed nuanced transcriptomic differences at the single cell level (Figure 6).

### Comparative Analysis of Heterogeneity Between Mandible and Femur Myeloid-Derived Suppressor Cells

**FIGURE 7:**
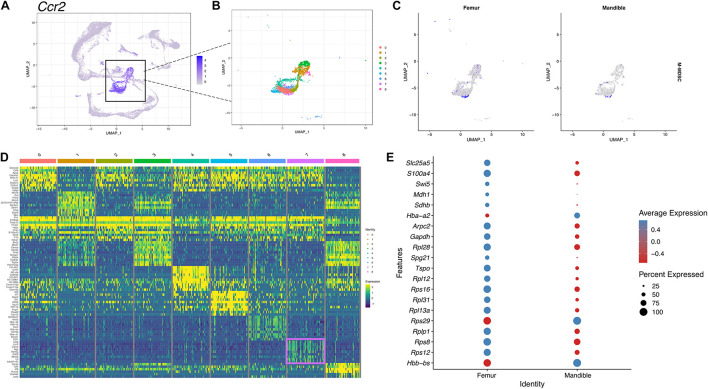
scRNA-seq of M-MDSCs in mBM reveals a transcriptional profile distinct from M-MDSCs in fBM. **(A)** M-MDSCs were identified as a subpopulation of the monocyte cluster marked by *Ccr2* expression. **(B)** The monocytic population was broken into nine distinct clusters. **(C)** Feature plots showing the M-MDSC composite score in femur and mandible. **(D)** The top ten most highly expressed genes in each cluster displayed in a heatmap showing expression on a log scale. The M-MDSC cluster seven genes are boxed in pink. **(E)** The top 20 differentially expressed genes in cluster 7 comparing the femur and mandible displayed in a dotplot, showing M-MDSC transcriptomic differences between the two anatomical locations.

Although we have provided an overall atlas of the immune cell, there may be limitations in detailing the sub-clusters of each immune cell. To further identify M-MDSCs, we first selected and displayed *Ccr2*-expressing monocyte clusters in UMAP (Figure 7A). The monocyte population was then further divided into nine distinct sub-clusters (Figure 7B) and cluster 7 was identified as the M-MDSC population based on expression of an M-MDSC specific gene panel (Figure 7C). There are notable differences in the M-MDSC population which also correspond to differences in transcriptomic profiles between the femur and the mandible (Figures 7D,E).

**FIGURE 8:**
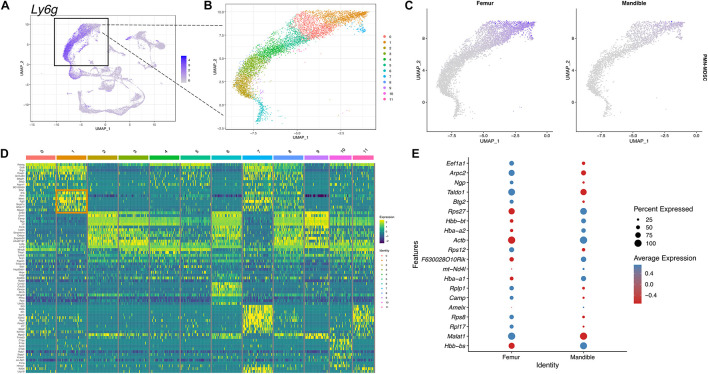
scRNA-seq of PMN-MDSCs in mBM reveals transcriptional profile distinct from PMN-MDSCs in fBM. **(A)** To identify PMN-MDSCs, the neutrophil cluster marked by *Ly6g* expression **(B)** was divided into 12 clusters. **(C)** A feature map of the PMN-MDSC composite genes show cluster 1 is enriched for PMN-MDSC genes. **(D)** Heatmap of the top 10 most highly expressed genes in each cluster. PMN-MDSC genes from cluster 1 are boxed in orange. **(E)** The top 20 differentially expressed genes in cluster 1 between femur and mandible displayed in a dotplot, showing PMN-MDSC specific transcriptomic differences.

To further identify PMN-MDSCs, we selected and displayed the neutrophil cluster expressing *Ly6g* gene in UMAP (Figure 8A). 12 different clusters were identified within the neutrophil population (Figure 8B). PMN-MDSCs were identified in cluster 1 based on the expression of the PMN-MDSC gene panel described in the materials and methods (Figure 8C). In the feature map, in both fBM and mBM, the gene expression of PMN-MDSC was most enriched in cluster 1 (Figures 8B,C). Consistent with the flow cytometry results in Figure 1A, the expression of PMN-MDSCs in mBM appeared to be lower (Figure 8C). We identified genes highly expressed in the PMN-MDSC cluster (Figure 8D), including *Fth1, Fgl2, Sirpb1c*, and *Mpeg1*, which were not included in our PMN-MDSC gene panel. This analysis also allowed us to pick up nuanced differences in gene expression within the PMN-MDSC sub-cluster between the femur and the mandible (Figure 8E).

## Discussion

Alveolar bone is the only osseous tissue continuously exposed to a complex array of microbial oral flora found within dental plaque biofilms. Indeed, periodontal homeostasis is maintained by a balanced host immune response to polymicrobial oral biofilms (Hathaway-Schrader and Novince, 2021). However, during disease states periodontal pathogenic microorganisms can penetrate the periodontal barrier to the reside in intimate contact with alveolar bone (Lamont et al., 2018). Therefore, the immune homeostasis of alveolar bone is more likely to be directly affected by microorganisms than any other osseous tissue. To highlight the need for alveolar bone to maintain homeostasis in this microbial laden microenvironment, stem/progenitor cells in the craniofacial region found in the mesenchymal compartment including dental pulp, periodontal ligament and alveolar bone have a higher osteoblastic capacity (Li et al., 2012). While other studies have suggested that neural crest-derived HSC harbor in the mandible (Jiang et al., 2016), in-depth studies of HSC have been lacking to better comprehend alveolar bone homeostasis in addition to understanding HSC in pathological conditions, including osteonecrosis and oral infection. The BM cell population may vary depending on the difference in developmental origin as well as the environmental niche. The differentiation and maturation of steady-state HPSCs is tightly regulated by intrinsic/extrinsic signals from a microenvironment called the niche. In this study, we found that immune cells in the mouse mandibular microenvironment have distinctive myeloid cell population differences with clearly different transcriptomic landscapes compared to femoral bone.

We observed that myeloid populations from murine mandibular bone marrow exhibit were consistently lower percentage of the total bone marrow population compared to long bone marrow myeloid cell populations. These data are consistent with published data indicating that monocytes (CD31^–^/Ly6C^*high*^) and myeloid blasts (CD31^+^/Ly6C^+^) were reduced in mBM (Faloni et al., 2011). Using flow cytometry to compare mandibular bone marrow and femur, we found that not only macrophage, but also M-MDSC populations, which are osteoclastic progenitors, are reduced in the mandibular bone. From scRNA-seq analyses, monocytes and neutrophils also showed lower percentages in mBM compared to fBM. The heterogeneity of myeloid cells between femur and mandible was additionally supported by detailed flow cytometry analysis of myeloid cell progenitor populations. Total GMP, a precursor of monoblast and myeloblast, was found in reduced percentage in mBM compared to fBM. Further analysis of macrophage progenitor (MP) populations indicated that this population was significantly reduced in the mandible compared to femur, indicating that the overall myeloid cell was deficient in mBM.

In this study, results between flow cytometry and scRNA-seq analyses were largely consistent except for observations made in the CMP population. Discrepancies in flow cytometry and scRNA-seq results have also been reported in previous study using bone marrow cells from healthy individuals (Oetjen et al., 2018). We suspect that the reason for these inconsistent results may be due to the small number within the CMP populations and the low number of distinct genes with overlapping transcriptional programs. This phenomenon is particularly likely to occur in cells from healthy mice that would not have expanded bone marrow populations due to infectious disease or tumor burden. Thus, fewer activated cells in this homeostatic system may limit the data set with fewer distinct differentiated cellular populations. Although most of the results were consistent, opportunities for further refinement should be highlighted as the use of single-cell technology expands.

Unexpectedly, we observed that M-MDSC isolated from the mandible had stronger immunosuppressive capacity than M-MDSC isolated from femur despite having fewer cellular percentage of this myeloid cell population. From a functional perspective, MDSCs isolated from normal healthy individuals have much lower immunosuppressive properties than those with pathological activation (Cheng et al., 2008; Huang et al., 2014; Heine et al., 2017). Although the mandibular bone is not a pathological condition *per se*, it is closer to a pathological condition than the femoral bone since the oral cavity is frequently exposed to foreign substances. As alveolar bone is bearing mechanical loads, whose force is two times higher than that of long bones (Ehrlich and Lanyon, 2002), coupled with a recent data indicating that mechanical stimulation promotes activation of myeloid-derived monocytes (Lin et al., 2021), implies that occlusal forces might lead to the difference of the immune microenvironment between alveolar bone and long bone. Thus, it is likely that M-MDSCs from mandibular bone are more activated and participating in immune surveillance, which may explain why M-MDSCs from mandible shows higher immunosuppressive capabilities than M-MDSCs from long bone. These data support an new and perhaps underappreciated function of MDSCs in the maintenance of alveolar bone homeostasis.

Myeloid-derived suppressor cells, as potent immunosuppressive cell populations, inhibit immune responses through complex mechanisms (Gabrilovich and Nagaraj, 2009; Ma et al., 2018; Consonni et al., 2019; Groth et al., 2019). The most widely accepted mechanism for MDSCs’ immunosuppressive ability by TGF-β, an immunosuppressive molecule, which can induce *Foxp3* expression in T cells (Chen et al., 2003; Fantini et al., 2004; Fu et al., 2004). Regulatory T cell also exhibits immunosuppression with various immunosuppressive molecules (Vignali et al., 2008). Based on this information, it is possible to postulate that mandibular M-MDSCs are more activated than in long bone M-MDSCs and induce regulatory T-cells by immunosuppressive molecules such as TGF-β to have stronger immunosuppressive properties. Indeed, we looked at mandibular M-MDSC transcriptomic profiles for *Tgfb* and other genes that would inhibit T-cell proliferation/function in our scRNAseq data set, but cell number and expression profile was too low to draw definitive conclusions (data not presented). This experimental limitation, due to number of mandibular bone M-MDSCs in healthy mice, necessitates additional studies to determine the immunosuppressive mechanisms within homeostatic mandibular bone.

Several studies have shown that osteoclasts, including the dynamics of osteoclastogenesis, exhibit different characteristics between mandibular and long bone (Aghaloo et al., 2010; Faloni et al., 2011). In experiments to differentiate progenitor cells into osteoclasts, it was reported that progenitor cells within mandibular and long bone undergo distinct differentiation processes. When we examined differentially expressed genes in the two groups for the CMP and GMP clusters, we observed that mandibular bone marrow myeloid progenitor populations have distinct transcriptomic programs. Several differences in gene expression profiling suggest that there could be differences in the transcriptional program of these osteoclastic progenitors in homeostatic mandibular bone. Indeed, early stage osteoclast differentiation in mandibular bone showed more osteoclast-like cells compared to long bone with increased multinucleation with increased number of large osteoclasts having greater than 10 nuclei in alveolar bone, although no differences in resorptive capacity were noted (Faloni et al., 2011).

In summary, we provide reference data sets for cell populations in the mandibular bone compared to long bone by multiple cell analysis approaches. Our study provides the possibility that the nature myeloid directed immune response and cellular fate in both oral health and disease may be due to the myeloid heterogeneity of the mandibular bone. These studies indicate the mandibular bone has a distinct transcriptional landscape compared to femoral bone with MDSCs that exhibit a more immunosuppressive phenotype. This highlights the need to investigate site-specific immune response in mandibular bone marrow populations during both homeostasis as well as pathological conditions, including periodontitis. Through better appreciation of the unique myeloid lineage microenvironment in the mandibular bone, more precise immunomodulatory targets for the treatment of oral-specific diseases are envisioned.

## Data Availability Statement

The datasets presented in this study can be found in online repositories. The names of the repository/repositories and accession number(s) can be found below: https://www.ncbi.nlm.nih.gov/geo/query/acc.cgi?acc=GSE179152.

## Ethics Statement

The animal study was reviewed and approved by the Animal Care and Use Committee protocol from the University at Buffalo using ARRIVE guidelines.

## Author Contributions

KHK performed the experiments and wrote the manuscript. NL, JB, EK, and LZ performed the experiments and edited the manuscript. SA and KLK designed the concept and all of the experiments and edited the manuscript. All authors contributed to the article and approved the submitted version.

## Conflict of Interest

The authors declare that the research was conducted in the absence of any commercial or financial relationships that could be construed as a potential conflict of interest.

## Publisher’s Note

All claims expressed in this article are solely those of the authors and do not necessarily represent those of their affiliated organizations, or those of the publisher, the editors and the reviewers. Any product that may be evaluated in this article, or claim that may be made by its manufacturer, is not guaranteed or endorsed by the publisher.
