# Supplementary figures and images for: Discovering Myeloid Cell Heterogeneity in Mandibular Bone – Cell by Cell Analysis
Source: Front Physiol. 2021 Sep 30;12:731549. doi: 10.3389/fphys.2021.731549 (PMC8514701; doi:10.3389/fphys.2021.731549)

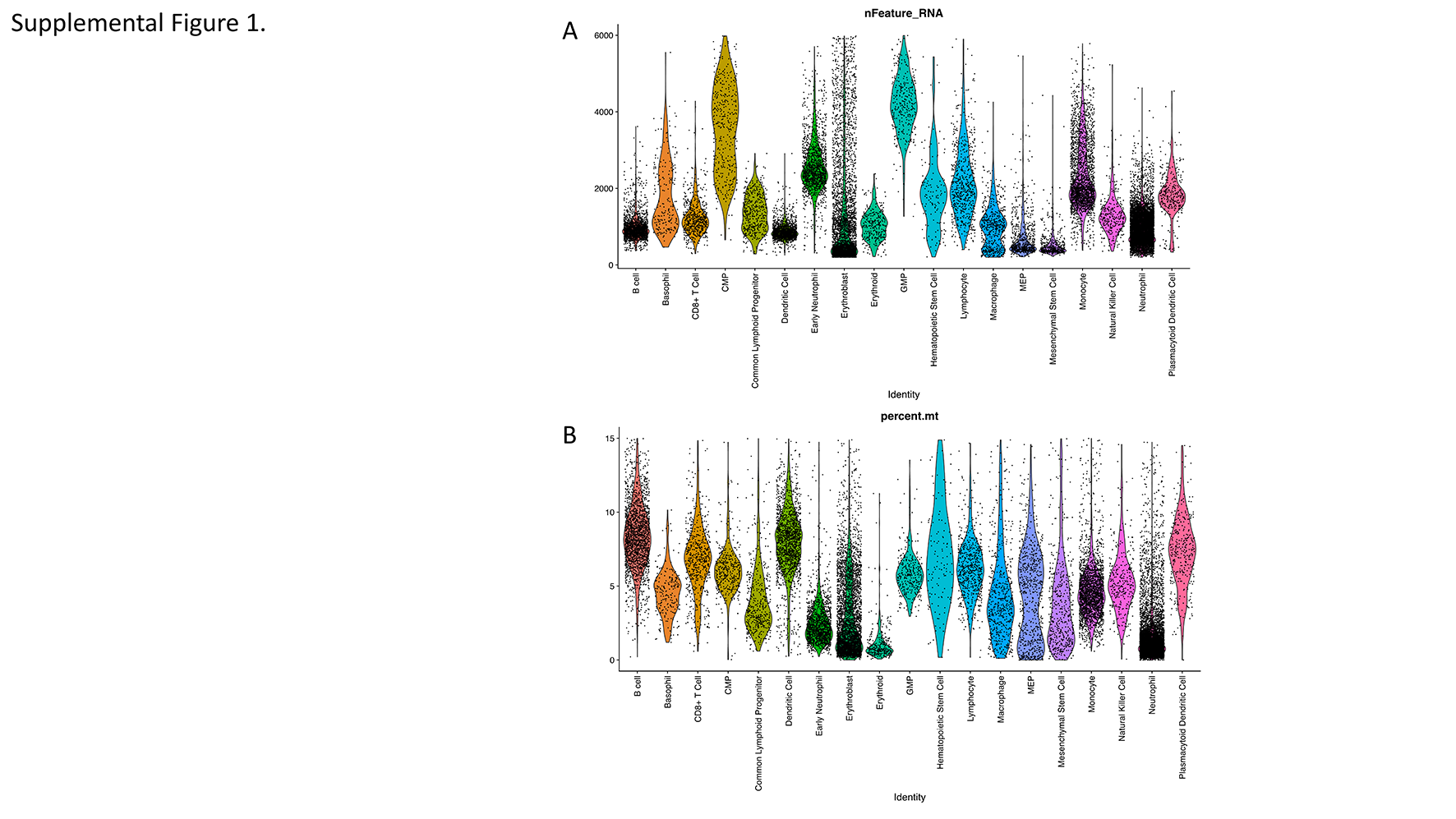

Supplement: Supplementary file 1 [file Image_1.tif]

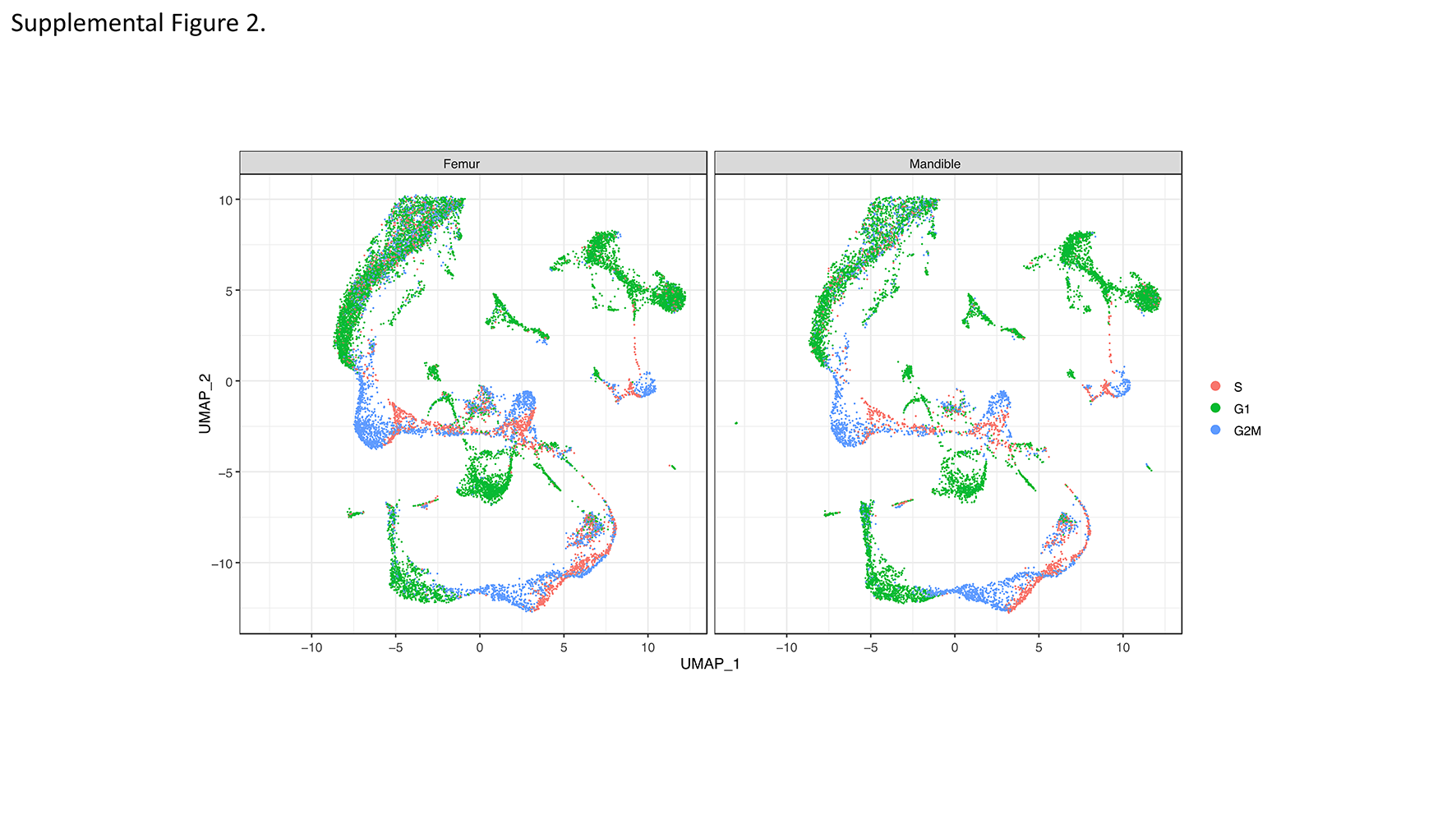

Supplement: Supplementary file 2 [file Image_2.tif]

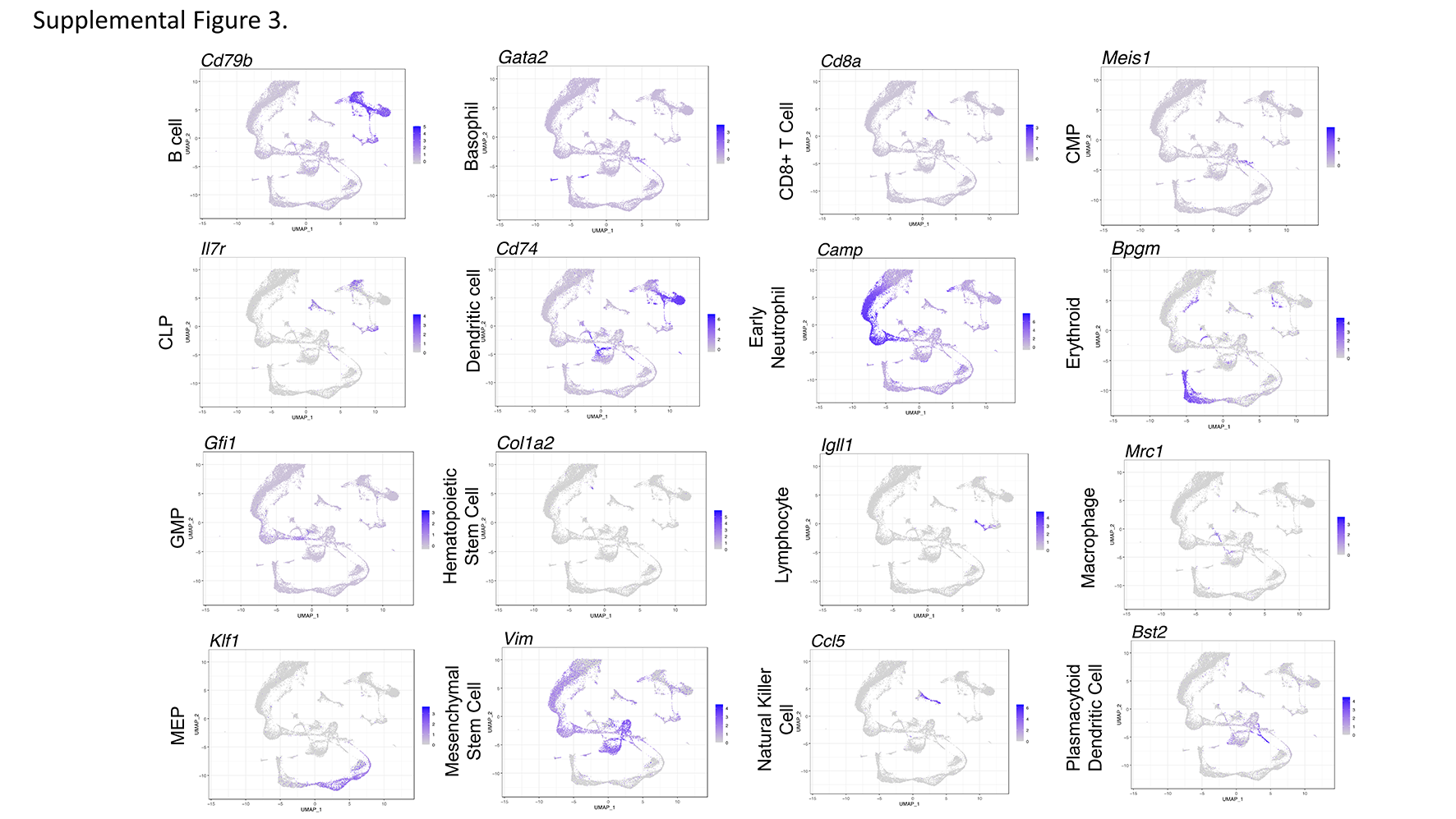

Supplement: Supplementary file 3 [file Image_3.tif]
